# A developed TaqMan probe-based qPCR was used to quantify the distribution of AMDV in various tissues of infected mink and its prevalence in northern China

**DOI:** 10.3389/fvets.2024.1498481

**Published:** 2025-01-07

**Authors:** Zaixing Yang, Yifan Li, Yuxuan Jiang, Jingqi Wu, Zhenhong Guan, Junwei Ge, Lili Zhao

**Affiliations:** ^1^State Key Laboratory for Diagnosis and Treatment of Severe Zoonotic Infectious Diseases, Key Laboratory for Zoonosis Research of the Ministry of Education, Institute of Zoonosis, and College of Veterinary Medicine, Jilin University, Changchun, China; ^2^Heilongjiang Provincial Key Laboratory of Zoonosis, College of Veterinary Medicine, Northeast Agricultural University, Harbin, China

**Keywords:** Aleutian mink disease virus, infection rates, tissue analysis, VP2, TaqMan qPCR

## Abstract

Aleutian mink disease (mink plasmacytosis) is a severe immune complex-mediated condition caused by the Aleutian Mink Disease Virus (AMDV), the most significant pathogen affecting mink health in the industry. Several studies have shown that AMDV epidemics can result in millions to tens of millions of dollars in economic losses worldwide each year. In this study, we developed a TaqMan probe-based real-time PCR technology (TaqMan-qPCR) for the specific, sensitive, and reproducible detection and quantification of AMDV in mink tissues by the VP2 gene, achieving detection limits as low as 1.69 × 10^1^ copies/uL of plasmid DNA and 8.50 × 10^−3^ ng/uL of viral DNA, and the established TaqMan-qPCR assay is 100 times more sensitive than PCR. Clinical samples of mink from different provinces showed a high prevalence of AMDV infection, 89.55% in Heilongjiang, 90.74% in Shandong, 80.23% in Hebei, 83.70% in Jilin, and 82.35% in Liaoning Province. Tissue distribution analysis showed that viral loads were generally high in all organs, especially in the mesenteric lymph nodes and spleen, and the virus was also detected in non-lymphoid tissues such as the brain, confirming the widespread distribution of AMDV throughout the body of mink. The established TaqMan-qPCR assay will become an important diagnostic tool for the prevention and control of AMDV, which is essential for disease management in mink populations.

## 1 Introduction

Aleutian mink disease virus (AMDV) is an icosahedral, envelope-less, self-replicating DNA virus 25 nm in diameter, that belongs to the Amdoparvovirus family (1, 2). It has a linear single-stranded genome of approximately 4.8 kb, comprised of two structural proteins (VP1 and VP2) and three non-structural proteins (NS1, NS2, and NS3) (1, 3). Within the genus Amdoparvovirus, the NS1 gene exhibits a higher degree of variability than the VP2 gene (4–6), and the VP2 protein is the central capsid protein of Amdoparvovirus and plays an important role in the pathogenicity and host range of Amdoparvovirus (7); therefore, the conserved region of the VP2 gene could serve as an ideal target for the detection of AMDV (8).

In 1940, the Aleutian mink virus was identified as a new cause of infectious peritonitis on mink farms in the United States and has since spread to all mink-producing nations (9). Mink Aleutian disease, also known as mink plasmacytosis, is a major infectious disease in minks and a significant global pathogen, leading to considerable economic losses (10). It impacts fertility and raises mortality, causing systemic problems in adult minks, including coat damage, reproductive issues (infertility, abortion, and reduced litter size), and interstitial pneumonia. Young minks are especially susceptible to pneumonia, which results in high death rates (11). Additionally, AMDV can infect other muskrat species such as weasels, skunks, and raccoons, and even non-muskrat species like bobcats. There are also reports of potential human infections (12). Epidemiological studies indicate that AMDV is prevalent on mink farms (13, 14). Despite its impact, research on AMDV is limited, particularly regarding its tissue distribution and pathogenicity, as well as its role in single or polymicrobial infections (15). Effective vaccines and treatments are lacking, highlighting the need for fast, accurate quantitative analysis techniques for better epidemiological monitoring and clinical management.

Currently, diagnosing AMDV infection in mink currently depends on pathological signs, PCR-based DNA testing, and antibody tests such as CIEP and ELISA (16–18). However, PCR is time-consuming and complex to analyze the results, as it requires gel electrophoresis for determination (19). Additionally, the production of antibodies usually takes 1 to 3 weeks after viral infection, which is later than the appearance of the virus (20, 21). Real-time fluorescent quantitative PCR technology (qPCR) offers high specificity, sensitivity, and relatively low cost, allowing for the rapid detection of viruses in various tissue samples, making it a vital tool in veterinary virology and disease management (22, 23). To date, only a few studies have focused on the detection of AMDV using qPCR. Li et al. established an EvaGreen-based qPCR method (24), Prieto et al. used a commercially available probe-based real-time PCR assay kit targeting the NS1 gene to study AMDV in the aquaculture environment, but the sequences of the primers and probes were not published (13, 14). Wu et al. developed a probe-based qPCR method based on the VP2 gene and Virtanen et al. developed a probe-based qPCR method based on the NS1 gene for the detection of AMDV in clinical samples (25, 26), but no studies have quantified AMDV in various tissues of infected mink. Furthermore, the TaqMan probe-based qPCR method is more specific than the EvaGreen-based qPCR method, because it uses probes that specifically target the pathogen being tested, making the TaqMan-qPCR less likely to yield false-positive results in co-infected samples. Given the economic significance of mink farming and the potential impact of AMDV on animal health, it is critical to establish reliable diagnostic methods that can accurately quantify viral load. In addition, understanding the dynamics of the virus in infected mink would also provide insight into transmission routes and inform control measures.

In this study, AMDV DNA was quantified by the TaqMan-qPCR, a standard curve was established and compared with conventional PCR. The TaqMan-qPCR analysis provided high sensitivity, specificity and reproducibility, which are essential for assessing viral exposure. The results showed that the TaqMan-qPCR was effective in detecting and quantifying AMDV in different tissues of clinically infected mink. Ultimately, we present a rapid and direct TaqMan-qPCR assay designed to be a precise and sensitive tool for AMDV detection.

## 2 Materials and methods

### 2.1 Viruses and samples

The AMDV LM strain, previously isolated by our team (GenBank accession no. KY680280), served as the positive control for both TaqMan-qPCR and conventional PCR assays. Other non-target virus controls used to test the specificity of the TaqMan-qPCR assay included Canine Circovirus (canineCV) isolated by our laboratory from Heilongjiang Province (GenBank accession No. MF797786). Mink calicivirus (MCV), rabies virus (RV), and canine adenovirus type 2 (CAV2) were sourced from Dr. Yongjun Wen (27), Dr. Jinying Ge (28), and Dr. Qian Jiang (29), respectively. Additionally, the vaccine strain of mink enteritis virus (MEV) and canine distemper virus (CDV) were procured from QiLu Animal Health Products, LTD., Shandong, China.

A total of 522 clinical samples from minks were collected from 37 farms in Heilongjiang, Jilin, Liaoning, Hebei, Shandong Provinces, China, spanning the period from May 2015 to December 2023. Clinical samples were defined based on key criteria for identifying mink as clinical cases of Aleutian Mink Disease Virus (AMDV). Observed clinical signs included behavioral changes (loss of appetite, decreased vitality), physical symptoms (temperature fluctuations, respiratory distress), neurological issues (poor coordination, seizures), and external lesions. Additionally, detailed health histories were collected to assess the clinical status and potential infection risk.

### 2.2 Primer design and synthesis

Twenty-two AMDV genome sequences sourced from GenBank were subjected to multiple comparisons using MEGA11 (MEGA Software, LLC, United States) to identify a highly conserved region within the VP2 segment of the genome; the design of essential primers (real-time quantitative PCR primers) and probes (Supplementary Figure S1) was subsequently conducted using Primer 5.0 (PREMIER Biosoft International, Palo Alto, California, United States), the classical PCR primer AMDVJ was derived from a previous study by Oie et al. (19), and was used as the primer for routine PCR assays in this study, as detailed in Table 1. The synthesis of these primers and probes was carried out by Comate Bioscience Co. Ltd., (Changchun, China).

**Table 1:** Primers and probes used in this study.

Name	Sequence (5′ to 3′)	Length (bp)	Position^a^
AMDVJ-F	CTTGTCACGCTACTAGAATGGT	693	2,587–3,279
AMDVJ-R	AGCTTAAGGTTAGTTTACATGGTTTACT		
AMDV-VP2-F	TAAGAAAAACGCAGAGATGAACA	108	3,434–3,541
AMDV-VP2-R	GGTCCTCCAGCAAAGTAACTACC		
AMDV-Probe	TACCAATATCCTGAATGGATAATACC		3,479–3,505

a Nucleotide positions are designated based on the AMDV strain LM gene (GenBank Accession No. KY680280).

### 2.3 DNA extraction

Based on the method of extracting DNA from mink clinical samples accroding to Cui et al. (30). In brief, 0.1 g of each tissue specimen was weighed, added to 500 μL of phosphate buffer, homogenized, freeze-thawed 3 times, and then centrifuged. The resulting DNA extract was purified using a Viral DNA Kit (Tran, Beijing, China) to eliminate any contaminants. The eluted DNA samples were then stored at −20°C until required.

### 2.4 Preparation of the standard plasmid

The 108 bp fragment of the VP2 gene was amplified from the DNA of AMDV strain LM using PCR with the AMDV-VP2-F and AMDV-VP2-R primers. The amplification products were then purified from the agarose gel using a Midi Purification Kit (Tiangen Biotech, Beijing, China). Subsequently, the purified products were ligated into the pMD19-T vector (Takara Biotechnology, Dalian, China) to create the recombinant plasmid pMDT-VP2. Following this, the pMDT-VP2 plasmid was introduced into *Escherichia coli* DH5α cells (TransGen Biotech, Beijing, China) and subsequently purified.

Using a plasmid miniprep kit (Axygen A Corning Brand, Suzhou, China) and confirmed through sequencing confirmation (Comate Bioscience, Changchun, China), the initial concentration of the target plasmid, pMDT-VP2, was found to be 51.75 ng/uL with a Nanodrop One spectrophotometer (Thermo Scientific, Wilmington, DE, United States). The plasmid’s copy number was calculated at 1.69 × 10^1^⁰ copies/uL, and it was stored at −20°C for future testing (31).

### 2.5 Establishment of a standard curve for the TaqMan-qPCR assay

To establish the standard curve, the standard plasmid pMDT-VP2 was serially diluted 10 times with elution buffer, ranging from 1.69 × 10^10^ copies/uL to 1.69 × 10^1^ copies/uL. The logarithm of the plasmid copy number was plotted against the measured cycle threshold (Ct) values. The Medtl System software automatically calculated the TaqMan-qPCR assay efficiencies, the standard curve, and the standard curve’s correlation coefficient. The number of copies per reaction was used to express the amount of DNA that was measured in each sample. The total volume was 10 uL comprising 5 uL of 2× Premix Ex Taq™ (Probe qPCR, TaKaRa, Dalian), 0.4 uL each of AMDV-VP2-F and AMDV-VP2-R primers (10 μmol/L), 0.2 uL of the AMDV-P probe (10 μmol/L), 1 uL of template, and 3uL of ddH2O.Using a Gentier 96E apparatus (Tianlong, Xian, China), fluorescence quantitative PCR was used to conduct the qPCR reaction in eight tubes. The procedure comprised incubation at 95°C for 1 min, followed by 40 cycles of 95°C for 10 s and 60°C for 15 s.

### 2.6 Validation of the TaqMan-qPCR assay

To verify the specificity of the method, qPCR was performed on seven viruses: AMDV, CanineCV, MCV, RV, CAV-2, MEV and CDV. This was done to rule out any qPCR cross-reactivities between AMDV and other diseases. AMDV-positive samples served as the positive control, while ddH2O acted as the negative control.

Dilutions of the PMDT-VP2 plasmid, from 1.69 × 10^10^ copies/uL to 1.69 x 10^0^copies/uL, were used to establish the TaqMan-qPCR assay’s detection threshold, with ultrapure water as a negative control. To evaluate the TaqMan-qPCR assay sensitivity for AMDV detection, diluted DNA from clinical tissue samples (ranging from 8.50 × 10^2^ ng/uL to 8.50 × 10^−4^ ng/uL) served as templates for both qPCR and conventional PCR, utilizing AMDVJ F and AMDVJ R primers in the conventional reaction. The diagnostic accuracy of the TaqMan-qPCR assay was validated with positively identified clinical samples confirmed by sequencing.

Three parallel tests were conducted using eight different dilutions of standard plasmid (10^9^–10^3^ copies/μL) amplification. Utilizing the formula CV = (SD [Ct value]/overall average [Ct value]) × 100, the coefficient of variation (CV) was computed to assess the TaqMan-qPCR assay’s intra- and inter-assay repeatability and stability.

### 2.7 Application of clinical samples

A total of 522 clinical samples including 75 serum sample, 402 different tissues of minks and 45 mink fecal samples were collected from 5 provinces, including Heilongjiang, Jilin, Shandong, Hebei, and Liaoning in China in 2015–2023. All samples were tested in in duplicate and independently using the established TaqMan-qPCR assay. Furthermore, these samples were also detected using conventional PCR simultaneously.

### 2.8 Assessment of viral load in mink tissues via the established TaqMan-qPCR assay

In this study, 12 naturally infected mink and 3 healthy control mink were collected to characterize the distribution of AMDV in mink tissues. The tissues, including liver, lung, spleen, heart, duodenum, jejunum, ileum, colon, brain, kidney, skeletal muscle, and mesenteric lymph nodes, were detected by the established TaqMan-qPCR assay to evaluate viral load. The viral load was quantified by the established TaqMan-qPCR assay using 1 μL of DNA per reaction. The DNA copy number of each sample was converted to copy number per gram by using the calculated Ct-value determined from the standard curve. All the samples were tested in triplicates.

## 3 Results

### 3.1 Standard curve

**Figure 1:**
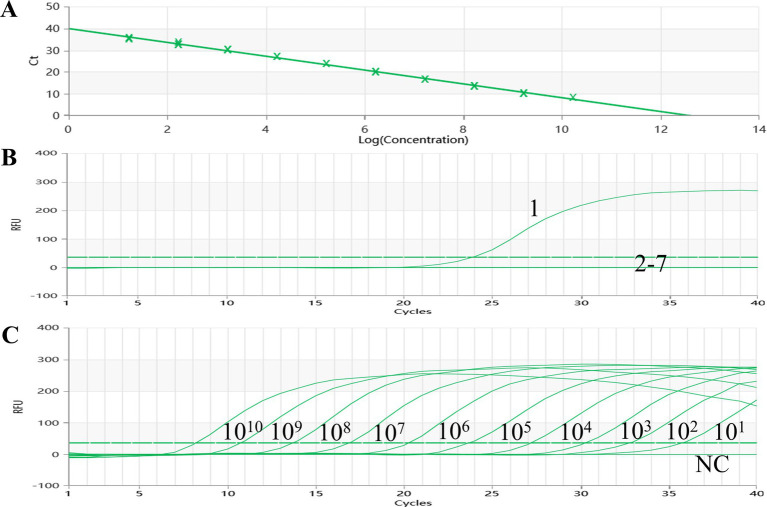
Amplification and standard curves of AMDV. **(A)** The standard curve (Slope: —3.164, Y-lntercept: 39.938, Efficiency: 107.036) was analyzed using Medtl System software. **(B)** Specificity of the qPCR was tested with six viral pathogens: l: AMDV LM strain DNA; 2—7: DNA and RNA samples of MEV, CDV, MCV, CAV2, RV,DogCV, and H20. **(C)** Amplification curves were generated using 10-fold dilutions of standard DNA from 10^10^ copies/gL to 10^1^ copies/11 L as controls.

A standard curve was meticulously constructed by plotting the logarithm of the plasmid copy number against the corresponding observed Ct values, utilizing a series of 10-fold dilutions of plasmids (Figure 1A). The linear relationship between the logarithm of the plasmid copy number and the Ct value exhibited an impressive correlation coefficient (R^2^) of 0.998, underscoring the robustness of the assay. Notably, the standard curve showcased a wide dynamic range spanning from 1.69 × 10^1^ to 1.69 × 10^10^ copies/uL, indicative of its sensitivity.

Furthermore, the established TaqMan-qPCR assay demonstrated a commendable amplification efficiency of 107.4%, affirming the reliability and accuracy of the designed test in quantifying the target DNA through the utilization of the standard curve.

### 3.2 Specificity, sensitivity, and reproducibility

The pMDT-VP2 plasmid was utilized as the AMDV DNA template in this experimental study. To assess the specificity of the established TaqMan-qPCR assay, seven distinct reactions were conducted, incorporating AMDV, CanineCV, MCV, RV, CAV-2, MEV and CDV, alongside a negative water control. Notably, reactions involving AMDV exhibited robust fluorescent signals, underscoring the specificity of the assay. In contrast, both the negative water control and the remaining six viral samples failed to yield any detectable signals (Figure 1B). The results unequivocally establish the accuracy and specificity of the established TaqMan-qPCR assay in precisely identifying the target virus, thereby eliminating the risk of cross-contamination or false-positive outcomes from non-target infections.

**Figure 2:**
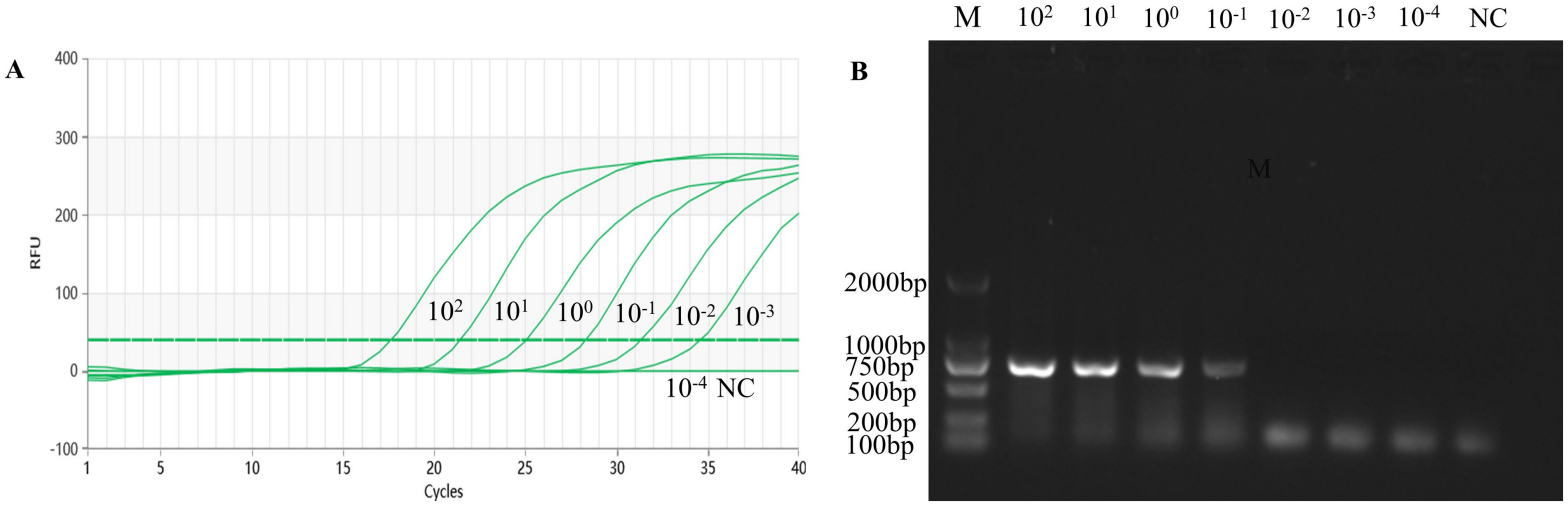
Sensitivity of the qPCR assay for the virus DNA ofAMDV clinical tissue samples. **(A)** The qPCR amplification curve. **(B)** Electrophoresis of conventional PCR reactions. The template concentrations of virus DNA ranged from 8.50 × 10^2^ ng/gl to 8.50 × 10–4 ng/gl. A negative control was included.

To assess the sensitivity of the TaqMan-qPCR assay reaction in this study, 10-fold serial dilutions of the standard plasmid were tested, which showed a qPCR detection limit of 1.69 × 10^1^ copies/uL for the standard plasmid DNA pMDT-VP2 (Figure 1C). Diluted DNA from AMDV clinical tissues (range 8.50 × 10^2^ ng/uL to 8.50 × 10^−4^ ng/uL) was used as a template by the established real-time fluorescence PCR method and conventional PCR. The detection limit was 8.50 × 10^−3^ ng/uL (Figure 2A), which was significantly better than the detection limit of 8.50 × 10^−1^ ng/uL for conventional PCR (Figure 2B).

In assessing the intra- and inter-assay reproducibility, eight dilutions of the standard plasmid pMDT-VP2 DNA were employed, ranging from 1.69 × 10^3^ copies/uL to 1.69 × 10^9^ copies/uL. The established TaqMan-qPCR assay exhibited exceptional reproducibility, as indicated by the intra-assay coefficient of variation (CV) values spanning from 0.20 to 0.76% and the inter-assay CV values ranging from 0.59 to 1.68% (Table 2). The narrow range of CV values underscores minimal variability within and between assays, further affirming the reliability and robustness of this assay.

**Table 2:** Repeatability validation of the TaqMan-qPCR assay.

DNA standard(copies/μL)	Intra-assay reproducibility			Inter-assay reproducibility		
	Mean Ct	SD	CV/%	Mean Ct	SD	CV/%
1.69 × 10^9^	10.20	0.04	0.40	10.33	0.07	0.68
1.69 × 10^8^	13.52	0.09	0.68	13.65	0.23	1.68
1.69 × 10^7^	16.70	0.07	0.40	16.90	0.10	0.59
1.69 × 10^6^	20.13	0.15	0.76	20.31	0.23	1.13
1.69 × 10^5^	23.81	0.05	0.20	23.96	0.15	0.63
1.69 × 10^4^	27.02	0.09	0.34	27.23	0.17	0.62
1.69 × 10^3^	30.23	0.19	0.62	30.37	0.33	1.09

### 3.3 Investigation of AMDV in test samples

The established TaqMan-qPCR assay was applied to clinically infected minks across 522 samples, revealing varying positive rates of AMDV across provinces: 89.55% in Heilongjiang, 90.74% in Shandong, 80.23% in Hebei, 83.70% in Jilin, and 82.35% in Liaoning Province (Table 2). In parallel, conventional PCR analysis on the same samples showed positivity rates for AMDV infection at 72.39, 77.78, 60.47, 68.48, and 70.59% in Heilongjiang, Shandong, Hebei, Jilin, and Liaoning Province, respectively (Table 3). Among the qPCR-positive samples (522 total), 366 were also positive using conventional PCR, while 74 samples tested negative with both methods, indicating higher sensitivity of the established TaqMan-qPCR assay over conventional PCR.

**Table 3:** Detection of AMDV in minks across various provinces.

		qPCR		Convention PCR	
Province	Number	Number positive/number tested	Positive rate (%)	Number positive/number tested	Positive rate (%)
Heilongjiang	134	120/134	89.55	97/134	72.39
Jilin	92	77/92	83.70	63/92	68.48
Liaoning	102	84/102	82.35	72/102	70.59
Hebei	86	69/86	80.23	50/86	60.47
Shandong	108	98/108	90.74	84/108	77.78
Total	522	448/522	85.82	366/522	70.11

### 3.4 Quantification of AMDV viral DNA from different tissues

In order to study the distribution of AMDV in infected mink, tissue samples from 12 positive mink and 3 control mink were determined by the established TaqMan-qPCR assay. 12 naturally infected mink were AMDV positive, and AMDV was detected in all of these 18 tissues. The viral loads of the 12 naturally infected mink are shown in Table 4 and Supplementary Table S1, AMDV is distributed in all tissues of infected mink. The number of AMDV DNA copies in different tissues of 12 naturally infected minks was shown below: heart (3.39 × 10^9^–3.39 × 10^4^ copies/g), skeletal muscle (4.72 × 10^8^–1.67 × 10^4^ copies/g), brain (6.04 × 10^9^–3.32 × 10^4^ copies/g), kidney (2.79 × 10^10^–2.12 × 10^5^ copies/g), duodenum (1.14 × 10^10^–1.44 × 10^4^ copies/g), jejunum (1.45 × 10^11^–6.24 × 10^5^ copies/g), lung (4.39 × 10^10^–1.59 × 10^5^ copies/g), liver (2.07 × 10^10^–4.42 × 10^4^ copies/g), colon (5.52 × 10^10^–1.92 × 10^5^ copies/g), mesenteric lymph nodes (1.73 × 10^12^–3.16 × 10^6^ copies/g), spleen (3.29 × 10^11^–5.55 × 10^5^ copies/g), oral swab(4.03 × 10^8^–1.75 × 10^5^ copies/g), nose swab (1.38 × 10^9^–6.61 × 10^4^ copies/g), eye swab (2.51 × 10^8^–3.94 × 10^4^ copies/g), body swab (7.79 × 10^8^–1.73 × 10^4^ copies/g),rectal feces (1.53 × 10^10^–1.42 × 10^5^ copies/g), blood (1.97 × 10^10^–1.57 × 10^3^ copies/g)and ileum (6.14 × 10^10^–7.97 × 10^4^ copies/g). In a descending order, the mean viral loads per tissue were as follows: mesenteric lymph nodes (2.95 × 10^11^ copies/g), spleen (4.58 × 10^10^ copies/g), jejunum (2.12 × 10^10^ copies/g), lung (1.10 × 10^10^ copies/g), ileum (9.21 × 10^9^ copies/g), colon (8.36 × 10^9^ copies/g), kidney (4.86 × 10^9^ copies/g) contained the highest viral loads, whereas liver (3.49 × 10^9^ copies/g), rectal feces (2.07 × 10^9^ copies/g), duodenum (1.93 × 10^9^ copies/g), blood (1.79 × 10^9^ copies/g), brain (8.61 × 10^8^ copies/g), heart (5.78 × 10^8^ copies/g), nose swab (1.38 × 10^8^ copies/g), body swab (1.18 × 10^8^ copies/g), skeletal muscle (8.46 × 10^7^ copies/g), oral swab (6.78 × 10^7^ copies/g), eye swab (5.68 × 10^7^ copies/g)had the lowest viral loads. Meanwhile, no AMDV was detected in the 3 healthy control minks.

**Table 4:** | AMDV DNA levels in the tissues of artificially infected minks measured by the TaqMan-qPCR assay.

**Virus load in infected minks (copies/g)**			
**Tissues**	**Scope**	**Average**	**Order**
liver	2.07 × 10^10^–4.42 × 10^4^	3.49 × 10^9^	8
lung	4.39 × 10^10^–1.59 × 10^5^	1.10 × 10^10^	4
spleen	3.29 × 10^11^–5.55 × 10^5^	4.58 × 10^10^	2
heart	3.39 × 10^9^–3.39 × 10^4^	5.78 × 10^8^	13
duodenum	1.14 × 10^10^–1.44 × 10^4^	1.93 × 10^9^	10
jejunum	1.45 × 10^11^–6.24 × 10^5^	2.12 × 10^10^	3
ileum	6.14 × 10^10^–7.97 × 10^4^	9.21 × 10^9^	5
colon	5.52 × 10^10^–1.92 × 10^5^	8.36 × 10^9^	6
brain	6.04 × 10^9^–3.32 × 10^4^	8.61 × 10^8^	12
kidney	2.79 × 10^10^–2.12 × 10^5^	4.86 × 10^9^	7
skeletal muscle	4.72 × 10^8^–1.67 × 10^4^	8.46 × 10^7^	16
Mesenteric lymph nodes	1.73 × 10^12^–3.16 × 10^6^	2.95 × 10^11^	1
rectal feces	1.53 × 10^10^–1.42 × 10^5^	2.07 × 10^9^	9
blood	1.97 × 10^10^–1.57 × 10^3^	1.79 × 10^9^	11
nose swab	1.38 × 10^9^–6.61 × 10^4^	1.38 × 10^8^	14
body swab	7.79 × 10^8^–1.73 × 10^4^	1.18 × 10^8^	15
oral swab	4.03 × 10^8^–1.75 × 10^5^	6.78 × 10^7^	17
eye swab	2.51 × 10^8^–3.94 × 10^4^	5.68 × 10^7^	18

## 4 Discussion

AMDV is one of three serious diseases that causes great economic loss in the mink industry with severe impacts on fertility, litter size and pelt quality (32). Despite the widespread impact of AMDV, serious knowledge gaps remain regarding the prevalence, tissue distribution, and pathogenic mechanisms of the virus, as well as a lack of effective vaccines and treatments to control the disease (33). Furthermore, AMDV in mink farms can cause environmental contamination of surfaces, furniture and equipment. The development of accurate diagnostic methods for use on mink ranches is therefore essential for the control of AMDV. The TaqMan-qPCR assay is used as a diagnostic tool because it is faster, more sensitive and allows for quantification. Therefore, the TaqMan-qPCR assay is a useful tool for studying the epidemiology of viral diseases (34), which makes it necessary in the detection of AMDV. This study developed and evaluated a specific and sensitive TaqMan-qPCR assay for the VP2 gene, enabling rapid detection and quantification of AMVD viruses in mink tissue samples and assessing the epidemiological situation in major mink breeding provinces in China.

Based on the published AMDV genome sequences in GenBank. Through our analysis of these sequences, we were able to identify a highly conserved region that exists within the AMDV VP2 genome. This identification allowed us to move forward with the design of specific primers and probes that are intended for use in qPCR detection. The results depicted in Figure 1A illustrate a favorable correlation between the copy number of the target DNA and the corresponding Ct-value. Specifically, we observed a slope of −3.155, indicating a strong relationship with an excellent correlation coefficient (R^2^ = 0.998) and high amplification efficiency (E = 107.4%). This demonstrates that our method is comparable to that of Wu et al. (25), while Virtanen et al. did not create a standard curve (26). These findings suggest that our established method for detection is not only highly efficient but may also represent a viable and suitable approach for diagnosing AMDV infections effectively (35, 30).

Specificity tests showed that the TaqMan-qPCR method developed in this study could reliably detect AMDV without cross-reacting with other viruses. The detection limit of this method was 1.69 × 10^1^ copies/uL of plasmid DNA, which was slightly lower than that of previous studies (25, 26). The lowest detection limit for AMDV DNA was 8.50 × 10^−3^ ng/uL, compared with 8.50 × 10^−1^ ng/uL for conventional PCR, indicating that our qPCR method is 100 times more sensitive. This high sensitivity, along with the elimination of post-PCR processing and the capacity for simultaneous detection and quantification, enhances its utility for disease surveillance (36). Moreover, the established TaqMan-qPCR assay showed reproducibility with a coefficient of variation (CV) between 0.20 and 1.68%, reflecting low variability and high reliability in both intraassay and inter-assay performance.

In this study, to evaluate the potential performance of the TaqMan-qPCR method, 522 clinical samples from mink farms in five provinces (Heilongjiang, Jilin, Liaoning, Hebei and Shandong) were tested by TaqMan-qPCR. The results showed that the prevalence of AMDV infection in mink farming in the country is still high, with an overall positive rate of 85.82 per cent in five provinces. According to Wang et al. (2014). The positivity rate using the CIEP assay was 81%, and the positivity rate in this study was slightly higher (8). Among them, Shandong province had the highest positive rate of 90.74%, Heilongjiang 89.55%, Jilin 83.70%, Liaoning 82.35% and Hebei 80.23%. In comparison, the total positive rate of the five provinces for routine PCR testing was 70.11%. Specifically, Shandong 77.78%, Heilongjiang 72.39%, Liaoning 70.59%, Jilin 68.48%, and Hebei 60.47%. This further demonstrated the superiority of the established TaqMan qPCR method in clinical detection.

Quantitative analysis of AMDV viral DNA in various tissues of infected mink revealed the distribution pattern of the virus. The results showed high viral loads in most tissues of AMDV-infected mink, with mesenteric lymph nodes and spleen being the highest (4.58 × 10^10^ copies/g – 2.95 × 10^11^ copies/g), followed by the jejunum and lungs (1.10 × 10^10^ copies/g – 2.21 × 10^10^ copies/g), which is in line with other local observations results (20, 37–39). This observation suggests the presence of active viral replication and viral shedding in these specific tissues, reflecting the dynamic interactions between the virus and host biological systems. Rectal feces had a moderate AMDV load, (2.07 × 10^9^copies/g), whereas oral swabs, nasal swabs and eye swabs had lower AMDV loads (5.68 × 10^7^copies/g – 1.38 × 10^8^copies/g), suggesting that feces may be the primary source of AMDV infection, these findings highlight the diagnostic power of TaqMan qPCR as an effective tool for determining the presence or absence of viruses, as well as the key role that these identified tissues may play as important reservoirs. These reservoirs are likely to be important contributors to the overall process of virus transmission between hosts (30).

Notably, these findings provide clues for uncovering information about the underlying mechanisms driving the pathogenesis of AMDV and its complex interactions with the host immune response (40). Understanding these dynamics is essential for the development of effective vaccines and targeted therapeutic strategies aimed at combating the virus and minimizing its impact (43). Moreover, it is noteworthy that the virus has also been reliably detected in non-lymphoid tissues, including the brain and skeletal muscle. This aspect significantly complicates our understanding of the virus’s pathogenicity, as it broadens the scope of tissues potentially affected by AMDV. Such findings not only highlight the virus’s far-reaching implications but also underscore its potential impact on broader wildlife health, raising additional concerns regarding ecosystem dynamics and the health of various species that may come into contact with infected individuals (42–44). These revelations indeed warrant further investigation into the complexities and consequences of AMDV infection.

This study provides valuable insights into the detection and quantification of AMDV in mink, however, there are some concerns. Although the study mentions that AMDV is prevalent in mink and shows high viral loads, there is a lack of insight into its specific pathological mechanisms and the nuanced impact of the host immune response. This is important because a deeper understanding of these mechanisms will be crucial for the development of effective vaccines and therapeutic strategies in the future. In addition, the sample size and geographic distribution involved in the study may also affect the positivity rate of AMDV infections, making larger studies necessary to obtain a more comprehensive and accurate assessment. Such extended studies will help to define more clearly the mode of transmission of the virus and its potential threat to the health of the mink population, thus providing a stronger scientific basis for relevant preventive and control measures. Moving forward, future research directions could include investigating the genetic diversity of AMDV strains circulating in mink populations, exploring host-pathogen interactions, and evaluating the efficacy of vaccination strategies in preventing AMDV infections (15). By addressing these unanswered questions, we can further advance our understanding of AMDV epidemiology and contribute to the development of effective control measures for this economically significant disease in minks.

## 5 Conclusion

In conclusion, this study successfully developed a highly sensitive and specific TaqMan probe-based qPCR assay for the detection and quantification of Aleutian mink disease virus (AMDV) in various tissues of infected minks. Given the significant economic impact of AMDV on the mink industry, understanding its prevalence and distribution is crucial for effective disease management. Our findings highlight the widespread presence of AMDV in multiple tissues, particularly with notably high viral loads in the mesenteric lymph nodes and spleen, suggesting these may serve as critical reservoirs for the virus. Additionally, the superior sensitivity of the TaqMan qPCR method compared to conventional PCR underscores its potential as a vital tool for epidemiological monitoring and clinical diagnosis of AMDV infections. By providing robust data on viral distribution and infection rates across different provinces in China, this research contributes to a broader understanding of AMDV’s pathogenicity and transmission dynamics. Future investigations should focus on exploring the genetic diversity of AMDV strains and the interactions between the virus and its host, which could inform the development of effective vaccines and therapeutic strategies. Continued surveillance and research are essential to mitigate the impact of AMDV on the mink industry and to enhance proactive disease control measures.

## Data Availability

The datasets presented in this study can be found in online repositories. The names of the repository/repositories and accession number(s) can be found in the article/supplementary material.
